# Robotic voluminous paraesophageal hernia repair: a case report and review of the literature

**DOI:** 10.1186/s13256-020-2347-6

**Published:** 2020-02-04

**Authors:** Nicola Tartaglia, Giovanna Pavone, Alessandra Di Lascia, Fernanda Vovola, Francesca Maddalena, Alberto Fersini, Mario Pacilli, Antonio Ambrosi

**Affiliations:** Department of Medical and Surgical Sciences, University of Foggia, Viale Pinto, 71122 Foggia, Italy

**Keywords:** Mini invasive surgery, Robotic surgery, Hiatal hernia, Giant or voluminous hiatal hernia, Nissen fundoplication

## Abstract

**Background:**

The treatment for sliding esophageal hernia with mild gastroesophageal reflux is usually conservative, but surgical treatment is recommended for refractory sliding esophageal hernia, paraesophageal hernia liable to prolapse, or paraesophageal hernia with ulceration and/or stenosis. Robotic surgery overcomes laparoscopic pitfalls by providing steady-state three-dimensional visualization, augmented dexterity with endo-wrist movements, and superior ergonomics for the surgeon.

**Case presentation:**

To investigate robotic paraesophageal hernia repair, a literature search was conducted using PubMed with the following key words: mini invasive surgery, robotic surgery, hiatal hernia, and Nissen fundoplication. We present the case of a 44-year-old Italian woman with a 20-year history of gastroesophageal reflux disease refractory to medical treatment, who underwent robotic Nissen fundoplication. In our center, we use the da Vinci® Xi™ Surgical System, which is an advanced tool for minimally invasive surgery.

**Conclusions:**

Various reports published in the literature suggested that the robot-assisted approach was effective and was associated with very low postoperative morbidity and was accompanied by satisfactory symptomatic and anatomical radiological outcomes during a follow-up period.

The robotic approach to paraesophageal repair is safe and effective with low complication rates. With increased experience, the operative time, length of stay, and complications decrease without compromising surgical principles.

## Introduction

Hiatal hernia is defined as the temporary or permanent migration of a portion or all of the stomach, or other viscera, into the mediastinum via a defect in the diaphragmatic crura, which normally define the esophageal hiatus. This is a very common clinical problem, affecting up to 60% of the adult population [1]. There are four types of hiatal hernias; however, the sliding hiatal hernia (type 1) is the most common and accounts for up to 95% of all hiatal hernias.

Type 1 hiatal hernias solely involve “sliding” of the gastroesophageal (GE) junction (GEJ) into the thoracic cavity. Types 2 to 4 hiatal hernias are true paraesophageal hernias (PEHs) and are classified based on the location of the GEJ as well as what has herniated into the thoracic cavity. A type 2 hiatal hernia has a GEJ in the normal anatomic position, but a portion of the stomach, most often the fundus, has herniated through the hiatus. Type 3, like type 2, has a portion of the stomach that has herniated through the hiatus, but also has an abnormal position of the GEJ in the thoracic cavity. Type 4 has an abnormal GEJ position like types 1 and 3 but another organ, most often a portion of the colon, has herniated into the thoracic cavity [2].

In the literature, PEH is mostly present among individuals aged 65 to 75-years old [3–5]. It is believed that most patients with PEH are asymptomatic. Symptoms can be caused by obstruction, GE reflux disease (GERD), bleeding, and iron deficiency anemia.

Obstruction at the GEJ or at the level of the pylorus can occur from intermittent twisting of the stomach along its long axis while herniating into the chest. If the GEJ is obstructed, the patient will complain of dysphagia and regurgitation, whereas gastric outlet obstruction produces nausea, vomiting, and epigastric or chest pain.

GERD is more common in sliding hiatal hernia but can occur in PEHs as well. In a series of 95 consecutive patients with GERD, those with a sliding hiatal hernia over 3 cm had a significantly shorter lower esophageal sphincter (LES) and greater reflux on pH monitoring than those with no sliding hiatal hernia or a sliding hiatal hernia < 3 cm [6]. Bleeding from the herniated fundus of the stomach owing to mucosal ulcers, known as Cameron lesions, can produce iron deficiency anemia.

Regardless of the mechanism of action, many patients with PEHs can have other nonspecific symptoms, such as postprandial chest pain, postprandial fullness, and shortness of breath. Patients with nonspecific symptoms can develop strangulation of the stomach from acute gastric volvulus, which constitutes a surgical emergency. In the management of those patients, a nasogastric tube cannot be placed into the stomach because patients retch but cannot vomit [7].

The treatment for sliding esophageal hernia with mild GE reflux is usually conservative. Surgical treatment is recommended for sliding esophageal hernia refractory to conservative treatment, PEH liable to prolapse, or PEH with ulceration and/or stenosis. In cases of PEH, prolapse may suddenly occur, causing complications such as gastrointestinal necrosis by strangulation, gastric perforation, or massive hemorrhage. A high mortality rate is associated with PEH with complications; therefore, surgical treatment for PEH with or without complications is recommended [8].

In this article, we present a case report of a 44-year-old woman with voluminous paraesophageal hiatal hernia treated with the robotic approach (Nissen fundoplication).

## Case report

A 44-year-old Italian woman was diagnosed as having hiatal hernia, confirmed with a new endoscopic examination in January 2018 associated with grade A esophagitis. She has history of refractory GERD, which was treated 15 years ago with esophagogastroduodenoscopy.

She reported several failed attempts of proton pump inhibitor therapy. In the last 3 years, she had various admissions to the emergency room due to violent epigastralgia associated with dyspnea and dysphagia, tachycardia, and vomiting.

Her past medical history includes hypertension well controlled by angiotensin-converting enzyme (ACE) inhibitors, asthma aggravated by tobacco smoking, and surgical treatment of endometriosis. She was subjected to gynecological and pneumological examinations during her hospital stay before surgery. There were no other surgical interventions abdominal or thoracic; there was no traumatic history. She had a familial history of arterial hypertension. She smoked approximately 20 cigarettes a day, consumed alcohol occasionally, she preferred carbonated drinks.

Physical and neurological examinations were not relevant, showing only a palpatory mild pain in the epigastrium and left hypochondrium, inferior liver margin at 1–2 cm below the ribs costal margin, and no other peculiar findings. On admission her blood pressure was 140/80 mmHg, breathing 18 breaths per minute, pulse 80 beats per minute, and temperature 36.5 °C. Complete blood count and liver and renal functions were within normal parameters.

Her imaging showed:

- chest X-ray – “a coarse opacity area with a conspicuous air–fluid level in the inferior mediastinum attributable to the hiatal hernia”;
- upper gastrointestinal tract radiography – “presence of voluminous hernia of part of the gastric body with cardia in place, paraesophageal hernia with rotation of the stomach.”

**Fig. 1:**
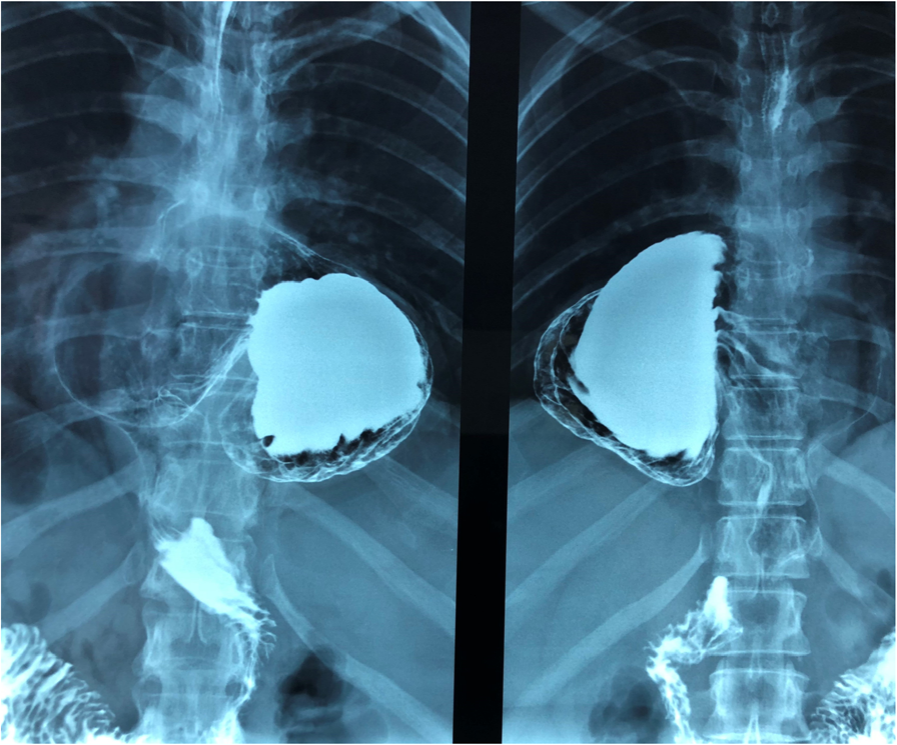
Preoperative upper gastrointestinal tract radiography

**Fig. 2:**
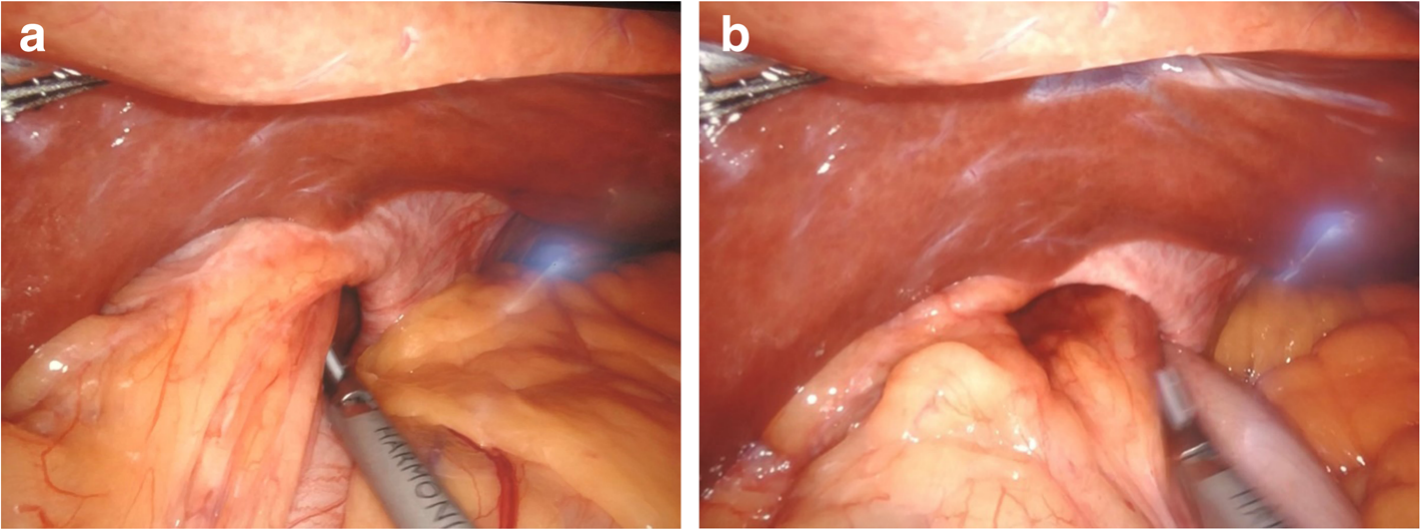
Voluminous hiatal hernia comprising 75% of the stomach, with the gastric bottom and the gastric body herniated through the esophageal hiatus (**a**) and rotated (**b**)

Therefore, she was diagnosed as having hiatal PEH type II, and in September 2018 she underwent robotic Nissen fundoplication (Fig. 1). The intraoperative findings showed a voluminous hiatal hernia comprising 75% of the stomach, with the gastric bottom and the gastric body herniated through the esophageal hiatus and rotated (Fig. 2 a, b).

### Surgical technique

In our center at the Department of Medical and Surgical Sciences of the University of Foggia, we use the da Vinci® Xi™ Surgical System, an advanced tool for minimally invasive surgery. This system acts as a natural extension of a surgeon’s eyes and hands, through a combination of cutting-edge robotics, three-dimensional stereoscopic vision, and intuitive human-interface controls.

**Fig. 3:**
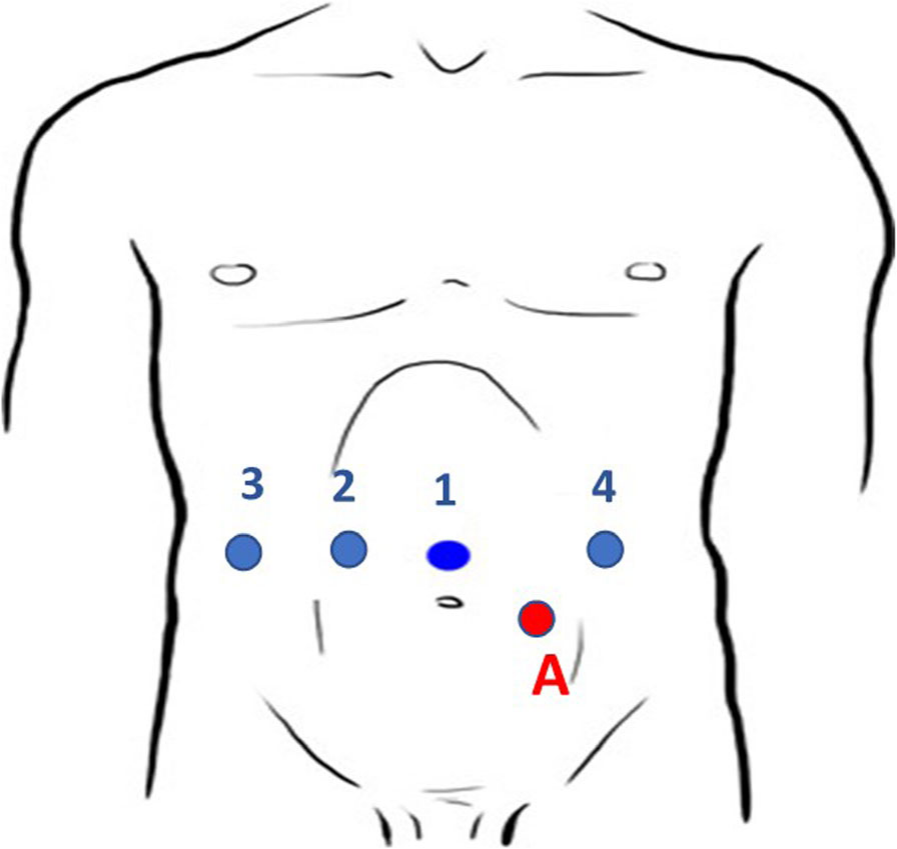
Ports position. *A* assistant

Our patient was positioned supine with both arms tucked in the anti-Trendelenburg position. The procedure was performed using five ports (Fig. 3). An 8 mm Opt iView trocar with a 0° scope was placed in the supraumbilical position to obtain peritoneal access under direct visualization, and a pneumoperitoneum was created. Two other 8 mm trocars were on the left side of her navel: one at the right another 8 mm and a 12 mm AirSeal access port for the assistant. After this, the robot was attached to the left shoulder of our patient.

The surgeon then started the dissection at the surgeon’s console. The hernia contents were reduced to expose the hiatus. The gastrohepatic ligament was moved, and the right crus was exposed. The procedure was started at the right crus, and the sac was bluntly separated from the mediastinal tissue, while dividing the sac circumferentially at the hiatal orifice. The short gastric vessels were moved to expose the left crus and complete the circumferential dissection, and this completely reduced the intrathoracic sac and moved any remaining contents into the abdomen.

An anterior crural repair was then performed using multiple interrupted polyfilament suture with intracorporeal knotting. A 360° Nissen fundoplication was performed, with placement of tubular drainage.

Then, the robot was undocked after removing the liver retractor under direct vision. Fascial layers were closed. All port sites were then closed with subcuticular stitches.

Our patient presented a postoperative course without complications and was discharged on the sixth postoperative day.

### Follow-up

**Fig. 4:**
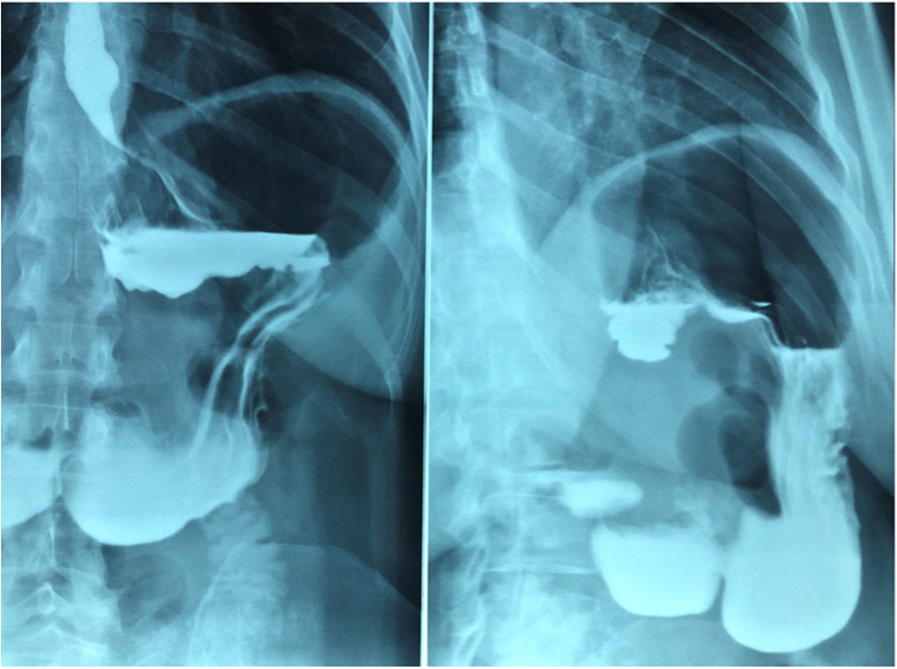
Postoperative upper gastrointestinal tract radiography

One month after surgery our patient underwent upper gastrointestinal tract radiography, which highlighted: “normal esophageal transit with normal aspect of the walls, cardia in place without evident refluxes. Regular canalization of the stomach, pylorus, and jejunal loops” (Fig. 4).

Six months after surgery we visited our patient, who denied nausea, vomiting, epigastralgia, and dysphagia and who noted a lifestyle improvement.

### Materials and methods

To investigate robotic PEH repair, a literature search was conducted using PubMed with the following key words: mini invasive surgery, robotic surgery, hiatal hernia, Nissen fundoplication. Only articles written in English were selected for primary review. The following data elements were extracted from articles that met the stated inclusion criteria: lead author, publication year, study design, inclusion and exclusion criteria, number of surgeries performed, morbidity, and mortality rates. Articles were excluded from the study if they focused on methods other than robotic surgery.

## Discussion

For over four decades, the management of PEHs has experienced a great deal of controversy. Surgeons have gone from watchful waiting to advocating elective repair even for asymptomatic patients due to the high mortality rates reported from mere observation, sometimes despite high operative risk [9, 10]. However, more recent literature has shown that the mortality rates for emergency PEH repair may not be as high as previously believed [11]. In fact, a study by Stylopoulos *et al.* demonstrated that the elective repair of completely asymptomatic patients may not be justified considering that the development of emergency symptoms was 1.16% per year [12]. Thus, symptomatic patients with an acceptable operative risk are recommended for repair.

This article presents the case of a 44-year-old woman with a 20-year history of GERD refractory to medical treatment with proton pump inhibitor, who underwent robotic Nissen fundoplication. In our center, we use the da Vinci® Xi™ Surgical System, which is an advanced tool for minimally invasive surgery. The data obtained with our study are in line with the literature.

Robotic-assisted surgery is slowly gaining popularity in general surgery, and numerous reports have been published on the safety and feasibility of robotics in procedures such as cholecystectomy, colorectal surgery, and, more recently, gastrectomy, and pancreatic surgery [13, 14].

In recent years, laparoscopic surgery has become the favored method for hiatal hernia repair because of its known advantages over open surgery, such as reduction of the physiological insult, reduction of postoperative pain, faster return of gastrointestinal function, faster recovery, shorter length of hospitalization, and superior visualization of the hiatal anatomy, which is crucial for mediastinal mobilization of the esophagus. However, although the laparoscopic approach has been demonstrated to be feasible and safe in several recent studies, patients with giant PEH are particularly challenging to manage.

Laparoscopic repair is currently considered standard treatment for symptomatic PEH in most academic centers. Although it is a technically challenging procedure, it has been proven to be safe and effective and is associated with excellent long-term patient outcomes in large reported series [15–17]. The procedure has the advantages of a minimally invasive approach, such as reduced postoperative pain, lower morbidity, and shorter hospital stay when compared to the open approach [18]. There are certain recognized pitfalls of laparoscopy, which include unstable video camera platform, limited motion (degrees of freedom) of straight laparoscopic instruments, two-dimensional imaging, and poor ergonomics for the surgeon [19]. These factors significantly increase the learning curve for complex surgical procedures. Robotic surgery overcomes these pitfalls by providing steady-state three-dimensional visualization, augmented dexterity with endo-wrist movements, and superior ergonomics for the surgeon. Laparoscopic repair is technically difficult in this subset of patients because of the presence of great anatomical distortion, which requires meticulous dissection of the hernia sac that must be accomplished with limited motion of rigid instruments and poor ergonomics [20–22]. Furthermore, various reports published in the literature (Table 1) suggest that there is a higher recurrence rate after the laparoscopic approach for giant hiatal hernia (GHH) repair than after conventional surgery [28–30].

**Table 1:** Comparison of reported series of paraesophageal hernia repair: laparoscopic and robotic studies

	*Outcomes*				
	*n*	Mean OT (*minutes*)	LOS (*days*)	Conversion (%)	Mortality (%)
Andujar *et al.* (2004) [15] (laparoscopic)	166	160	3.9	1.2	0
Draaisma *et al*. (2008) [23] (robotic)	40	127	4.5	0	0
Braumann *et al*. (2008) [24] (robotic)	14	134	6.5	0	0
Galvani *et al*. (2016) [25] (robotic)	61	186	1.7	0	0
Gehrig *et al*. (2013) [21] (robotic)	12	172	7.8	8	0
Vasudevan *et al*. (2018) [26] (robotic)	28	83	2.8	0	3.4
Morelli *et al*. (2015) [27] (robotic)	6	182	5	0	0

*OT* operative time, *LOS* length of stay

Andujar *et al.* (2004) [15] analyzed a total of 166 patients with a mean age of 68 years who underwent the laparoscopic approach. PEH were type II (*n* = 43), type III (*n* = 104), and type IV (*n* = 19). Mean operative time (OT) was 160 minutes. Fundoplications were Nissen (127), Toupet (23), Dor (1), and Nissen–Collis. Fourteen patients underwent a gastropexy. One patient required early reoperation to repair an esophageal leak. Reoperation was required in ten patients (6%): two for symptomatic recurrent PEH (1.2%), four for recurrent reflux symptoms (2.4%), and four for dysphagia (2.4%).

Draaisma *et al.* (2008) [23] suggested that the robot-assisted approach was effective and associated with very low postoperative morbidity and was accompanied by satisfactory symptomatic and anatomical radiological outcomes during a follow-up period of at least 1 year. In this study, median operating time was 127 minutes, and median blood loss was 50 ml. Intraoperative complications occurred in two patients (5%), and early postoperative complications occurred in five patients (12.5%). Furthermore, three patients had to be reoperated during 30-day follow-up (7.5%). No patients died, and the median hospital stay was 4.5 days.

In a pilot study of 14 patients undergoing robot-assisted hiatal hernia repair, Braumann *et al.* (2008) [24] concluded that robotic hiatal hernia repair is feasible and safe; in this study, the population consisted of 280 elective patients who were submitted to a variety of robot-assisted laparoscopic or thoracoscopic surgery. Out of these, 14 patients with a PEH were operated with the da Vinci® Surgical System. Average operating time was 134 minutes, and the average hospital stay 6.5 days. There were no intraoperative surgical-related complications owing to the telerobotic system, and the patients’ postoperative courses were uneventful. No specific robotic surgery-related complication was detected.

In the largest series to date (61 patients), Galvani *et al.* (2016) [25] did note significantly decreased OT, blood loss, and length of hospital stay (LOS) as the surgeon’s experience improved from the 16th to the 22nd case, respectfully. We note that their reported operating time of 186 minutes and LOS of 1.7 days vary significantly from our data. Further study is required from robotic centers of excellence to delineate standards of practice in this regard.

Gehrig *et al.* (2013) [21] noted in their case–control study that robotic PEH repair yielded a shorter hospital stay and fewer complications when compared to open repair but was similar in outcomes and OT to laparoscopic repair. They compared 12 patients who underwent paraesophageal hiatal hernia repair using a robot with 17 and 13 patients who underwent conventional laparoscopic and open repair, respectively.

Vasudevan *et al.* (2018) [26] in a retrospective cohort study of 28 consecutive patients who underwent robotic PEH repair concluded that the mean OT, including the robot docking time, was 83.6 + 24 minutes. The average LOS was 2.8 ± 1.9 days. There were no conversions to open or laparoscopic procedures. Postoperative complications were noted in three patients (10.7%), including one mortality (3.4%). One symptomatic recurrence (3.4%) was noted during the 12-month follow-up period.

In a 3-year prospective assessment, Morelli *et al.* (2015) [27] analyzed six patients with giant hiatal hernias who underwent robotic repair using the da Vinci Surgical System. The average operating time was 182 minutes. The average admission was 6 days. No patient required reoperation for recurrence of the disease, and all claimed the absence of postoperative symptoms.

As we have seen in the literature, even our case report is in line with these data, with an operating time of 140 minutes, and there were no conversions or deaths; the LOS was slightly higher than the studies taken into consideration: 9 days, 6 of which were postoperative. This was due to the need to subject our patient to gynecological, gastroenterological, and pneumological examinations in relation to her comorbidity before the repair of hiatal hernia.

## Conclusions

We conclude that the robotic approach to paraesophageal repair is safe and effective with low complication rates. With increased experience, the OT, LOS, and complications decrease without compromising surgical principles. The open approach to PEH repair is becoming obsolete because of the associated high morbidity. Future studies with larger numbers of patients and prospective randomized control trials are needed to demonstrate the durability of this procedure compared with the current laparoscopic approach.
